# Influence of the Culture Conditions on *Camellia sinensis* Cell Cultures

**DOI:** 10.3390/foods13152461

**Published:** 2024-08-04

**Authors:** Pilar Esteban-Campos, Pilar Vela, Raquel Rodríguez-Solana, José Ignacio López-Sánchez, Carmen Salinero, Efrén Pérez-Santín

**Affiliations:** 1 Estación Fitopatolóxica Areeiro, Deputación de Pontevedra, Subida á Carballeira, 36153 Pontevedra, Spain; pilar.esteban@depo.es (P.E.-C.); pilar.vela@depo.es (P.V.); carmen.salinero@depo.es (C.S.); 2 Department of Agroindustry and Food Quality, Andalusian Institute of Agricultural and Fisheries Research and Training (IFAPA), Rancho de la Merced Center, Carretera Cañada de la Loba (CA-3102) Km 3.1., SN, 11471 Jerez de la Frontera, Spain; raquel.rodriguez.solana@juntadeandalucia.es; 3 MED—Mediterranean Institute for Agriculture, Environment and Development, Faculdade de Ciências e Tecnologia, Universidade do Algarve, Campus de Gambelas, 8005-139 Faro, Portugal; 4 CHANGE—Global Change and Sustainability Institute, Faculdade de Ciências e Tecnologia, Universidade do Algarve, Campus de Gambelas, 8005-139 Faro, Portugal; 5 Escuela Superior de Ingeniería y Tecnología (ESIT), Universidad Internacional de la Rioja—UNIR, Avenida de la Paz, 137, 26006 Logroño, Spain; joseignacio.lopez@unir.net

**Keywords:** *Camellia sinensis* cell culture, tea, catechins, biomass productivity, elicitors, growth regulators

## Abstract

Since the last century, it has been shown that dedifferentiated cells of *Camellia sinensis* can produce catechins and other secondary metabolites under in vitro conditions, with potential applications in the cosmetic, pharmaceutical and food industries. In this work, cell suspension cultures of a *C. sinensis* cell line (LSC-5Y) were established in a liquid medium in order to optimize the biomass productivity, catechin monomer (GC, EGC, C, EC, CG, and ECG) and alkaloid (TB and CAF) productivity. The following factors were evaluated: concentration of growth regulators (BA and IBA), inoculum size, age of the cell line, light exposure, and effect of biotic elicitors (MeJA and extracts of *Ciborinia camelliae*). GC, EGC, and ECG increased approximately 1.80-fold when the auxin IBA concentration was increased from 0.1 to 2.0 mg/L. In addition, better productivity of EGC, C, EC, and CAF was achieved by using inoculum densities between 50 and 100 g/L. Although lower inoculum densities (25 g/L) showed a higher growth rate (0.20 d^−1^), the use of inoculum densities higher than 25 g/L favors a 2–4-fold increase in total catechin (TC) productivity, with maximum productivity being reached after 21 days of culture. However, the cell line showed instability in TC productivity: in the short term (in three successive subcultures), the coefficient of variation was 32.80%, and catechin production capacity was 2.5 years with maximum productivity at 0.5 years. Finally, it was observed that ethanol, used as an elicitor solvent, has a strong elicitor effect capable of increasing the accumulation of catechins up to 5.24 times compared to the treatment without an elicitor.

## 1. Introduction

Tea, the second most consumed beverage after water, is made from *Camellia sinensis* (L.) O. Kuntze (*Theaceae* family). All the different types of tea are produced from the tender shoots and leaves of the *Camellia sinensis* plant [1,2]. Global tea production reached 6.7 million tons in 2022, with an estimated market value of around USD 122 billion, with the main producing countries being China, India, Kenya, and Sri Lanka [3]. Due to the economic importance of this beverage, very extensive studies have been carried out to determine the profile and content of secondary metabolites in the leaves of *C. sinensis*, as well as their beneficial properties for human health [1]. In this way, a large number of bioactive compounds have been identified with applications in the pharmaceutical, cosmetic, and food industries, due to their antioxidant, antidiabetic, anticarcinogenic, photoprotective, or relaxing properties, among others [2]. The most studied secondary metabolites in tea leaves are the catechins, because they represent between 10% and 30% of the dry weight of the leaf and because of their high antioxidant capacity [1,2,4]. The main catechins found in tea leaves are epicatechin (EC), epigallocatechin (EGC), epicatechin gallate (ECG), epigallocatechin gallate (EGCG), catechin (C), gallocatechin (GC), catechin gallate (CG), and gallocatechin gallate (GCG). Each type of catechin has different antioxidant properties and confers different organoleptic properties to tea infusions [5]. The second most studied type of secondary metabolites in tea buds are alkaloids, such as caffeine (CAF) and theobromine (TB), which can make up to 3% of the dry weight of the leaf [6,7]. Tea buds are also rich in the non-protein amino acid L-theanine (5-*N*-ethylglutamine, Thea), which represents between 1 and 4% of the dry weight of the leaf [7], has a relaxing effect, and stimulates the immune system [2].

The diverse properties of secondary metabolites found in tea have led to their extensive use, particularly in the cosmetic, nutraceutical, and food industries, where extracts from tender shoots of field-grown tea plants are commonly used. However, the variability of secondary metabolite content in tea leaves due to environmental factors has prompted the exploration of alternative production methods based on plant biotechnology [1,8,9]. This is because these metabolites play an ecological role as secondary metabolites, protecting the tea plant against abiotic stresses (high light intensity, UV radiation, extreme temperatures, or drought) or biotic stresses (such as plant infection by insects or fungi) [6]. The first dedifferentiated tea cell cultures were developed in 1969 [10]. The aim of these cultures was to produce secondary metabolites under controlled conditions in order to eliminate the culture factors that affect the productivity of these compounds under field conditions. For this purpose, Forrest (1969) [10] studied the metabolism of polyphenols in tea stem callus and demonstrated the ability of these calluses to synthesize simple catechins (C and EC) [10]. In the 1990s, Orihara and Furuya [11] studied the ability of stem tea callus to produce purine alkaloids and the amino acid L-theanine. More recent studies in the 21st century have focused on optimizing the growth of dedifferentiated cell lines and hairy root lines of *C. sinensis* in liquid medium, as well as studying the production capacity of these cell lines for monoterpenes, catechins, caffeine, and L-theanine [5,12,13,14,15].

Studies by Shibasaki-Kitakawa and Muthaiya [12,14] have succeeded in producing between 144 and 228.5 mg/g dry weight (DW) of total flavonoids or total catechins by tea cell suspension cultures (CSCs), achieving high productivities of 1.50 g/L of flavonoids. Several of these studies focused on how culture conditions (salt medium, type, and concentration of plant growth regulators, pH medium, inoculum density) affect biomass productivity in different tea cell lines but not on the *C. sinensis* flavonoid profile/content and specifically the catechin yield (major plant compound) [5,12,13,14,15]. On the other hand, the effect of other cultivation factors such as cultivation time (days), the use of light treatment as an abiotic elicitor and the use of biotic elicitors on the production of catechin monomers in tea cell lines have not been previously studied. For this reason, the production capacity of catechins by tea CSCs has mostly been studied using non-directed analytical techniques such as spectrophotometric techniques based on the quantification of total phenolic compounds or total flavonoids [14,15,16]. Only in the study of Muthaiya et al. [12] was the variation of monomeric catechins using liquid chromatography investigated as a response to the addition of different precursors.

In this context, this study evaluated the influence of different cultivation factors [plant growth regulator (PGR) concentration, inoculum size, light exposure, and the addition of biotic elicitors] on biomass productivity and metabolite production [total catechin (TC) and monomeric catechins] by *C. sinensis* cell lines. The aim was the long-term, controlled implementation of an industrial-scale biological process with high metabolite production.

## 2. Materials and Methods

### 2.1. Chemicals and Reagents

Chemicals for PGRs: 6-benzylaminopurine (BA), dimethylallylamino purine (2-iP), indole-3-butyric acid (IBA), kinetin (Kn), and trans-zeatin (tZ) were purchased from Duchefa Biochemie (Haarlem, Netherlands) and 2,4-dichlorophenoxyacetic acid (2,4-D) from Sigma-Aldrich (Merck, Darmstadt, Germany). Acetic acid, acetonitrile, methanol, and water (HPLC grade) were supplied by Fisher-Scientific (Geel, Belgium). The standards epicatechin (EC), epigallocatechin (EGC), epigallocatechin gallate (EGCG), epicatechin gallate (ECG), catechin (C), gallocatechin (GC), gallocatechin gallate (GCG), and catechin gallate (CG) were purchased from Extrasynthese (Genay, France). Theophylline (TF) was obtained from Acros Organics (Geel, Belgium) and caffeine (CAF) and theobromine (TB) from Fisher-Scientific (Geel, Belgium). Methyl jasmonate (MeJA) and gallic acid (GA) were purchased from Sigma-Aldrich (Merck, Darmstadt, Germany); see Table 1.

### 2.2. Plant Material and Callus Induction

**Figure 1:**
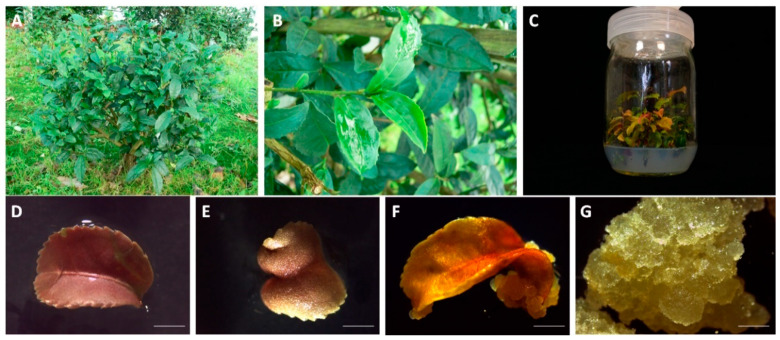
(**A**) Field plant *C. sinensis* clone EFA-5. (**B**) Tender shoot and node segments of tea plant. (**C**) Tea seedling established under in vitro conditions. (**D**) Leaves sown on day 0, (**E**) 11, and (**F**) 51, and (**G**) callus established after more than 3 years of subculture. Scale bar is 5 cm.

Nodal segments of *Camellia sinensis* clone EFA-5, grown in the field, were harvested from the camellia collection at the Estación Fitopatolóxica Areeiro (NW Spain, N 42° 24′ 22.89″, W 8° 40′ 24.64″, altitude about 70 m) for the initiation of in vitro seedlings (Figure 1A,B). This EFA-5 cultivar was 5 years old and was grown under semi-shaded conditions with sprinkler irrigation and controlled fertilization. Tea seedlings were established in Woody Plant Medium (WPM) supplemented with 4 mg/L BA, 0.1 mg/L IBA, 3% sucrose (sac), and 0.56% Plant Propagation Agar (PPA) (Condalab, Madrid, Spain) at 25 ± 2 °C with a photoperiod of 16 h of light and 8 h of dark (cool white fluorescent lamps, 40 μmol·m^−2^·s^−1^), and subcultured every 6 weeks (Figure 1C–E). Seedling propagation was carried out under the same culture conditions using WPM supplemented with 2 mg/L BA, 2 mg/L 2-iP, 2 mg/L tZ, 0.01 mg/L IBA, 3% sac, and 0.56% PPA [17].

For the induction of tea callus (Figure 1F), leaves from the in vitro seedlings were excised into 1 cm^2^ fragments and cultured on Gamborg B5 medium (Duchefa Biochemie, Haarlem, Netherlands) as previously described by Wang et al. [18], with some modifications. The medium contained 0.1 mg/L Kn, 0.5 mg/L 2,4-D, 0.8 g/L glutamine (Gln), 0.1 g/L serine (Ser), 3% sucrose (sac), and 0.56% PPA, and the cultures were maintained at 25 ± 2 °C in the dark. The resulting callus (Figure 1G) was transferred to CLC/Ipomoea medium, Embryo Development (EP), or Ep medium (Duchefa Biochemie, Haarlem, The Netherlands) supplemented with 2 mg/L BA, 0.1 mg/L IBA, 0.8 g/L Gln, 0.1 g/L Ser, 3% sac, and 0.56% PPA under the same culture conditions.

### 2.3. Selection and Screening of LSC-5 Callus Lines under Light Treatment

Cell suspension cultures of the *C. sinensis* line from EFA-5 (callus line LSC-5) were subcultured for 4 years in the same Ep medium described in the previous section and under the same culture conditions. After this period, 3 plates of 8 replicates each were cultured for 6 months in light conditions (cool white fluorescent lamps, 40 μmol·m^−2^·s^−1^), with a photoperiod of 16 h of light and 8 h of darkness. After this period, two cell lines were selected based on the basis of their appearance and designated as LCS-5Y, for its yellow color, and LCS-5R, for its purple-red color. Both lines were subcultured every 28 days for one year at 25 ± 2 °C and in total darkness in the same Ep medium, and the levels of target metabolites (catechin monomers and alkaloids) were analyzed by HPLC after 28 days of culture.

### 2.4. Establishment of Tea CSCs

LSC-5Y and LSC-5R callus lines (2.5 g per flask) were inoculated into 50 mL Erlenmeyer flasks (VWR) sealed with PTFE membrane plugs (0.2 µm pore diameter) to facilitate gas exchange. The culture volume was 30% of the flask capacity and consisted of the same Ep medium used for callus culture, but without PPA. Every 31 days, the fresh cell mass was harvested, and the culture volume was gradually increased by transferring the cells to larger Erlenmeyer flasks (100, 150, and 500 mL of capacity). Tea CSCs grown in 500 mL flasks were subcultured every 28 days using a fresh cell biomass equivalent to 25% of the culture volume as inoculum (50–70 g/L of inoculum size). Cultures were maintained under orbital shaking at 150 rpm and 25 ± 2 °C, with a light intensity of 4.5 µmol·m^−2^·s^−1^ and a photoperiod of 16 h of light and 8 h of dark. A 15-day-old LSC-5Y stock CSC was used to initiate subsequent optimization tests of culture conditions and elicitation treatments in 125 mL flasks, maintaining the same medium composition and culture conditions of shaking, light, and temperature as previously described.

### 2.5. Effect of PGR Concentration, Inoculum Size, and Cell Line Age on Biomass and Metabolite Productivity

A series of experiments were carried out on LSC-5Y CSC to investigate the effect of PGR concentration and inoculum size on biomass and target metabolite productivity. To study the effect of PGRs, three concentrations of the cytokinin BA (1.0, 2.0, and 3.0 mg/L) in Ep medium supplemented with 0.1 mg/L of the auxin IBA and four concentrations of IBA (0.1, 0.5, 1.0 and 2.0 mg/L) in Ep medium supplemented with 2 mg/L BA were examined. Two low inoculum densities on a fresh weight (FW) basis (25 and 50 g/L) and two high inoculum densities (100 and 150 g/L) were selected, and the experiment was carried out over three consecutive months to assess the effect of culture age and hence the short-term stability of the cell line. For this purpose, the coefficient of variation of TC productivity was analyzed using the following formula: (standard deviation/average) × 100%.

In addition, the growth kinetics of tea CSC were evaluated at low (25 g/L) and high (100 g/L) inoculum densities in order to assess the biomass productivity and cell viability at intervals of 0, 7, 14, 21, 28, and 35 days. For all tests, the culture time was 21 days (except for determination of growth kinetics) and an initial inoculum density of 25 g/L was set, except for the inoculum size assays.

### 2.6. Long-Term Stability of TC Production by Tea CSCs

From the stock mother culture of LSC-5Y CSC established in the 500 mL Erlenmeyer flask under the culture conditions previously described and which was used to start the rest of the studies of this research, the productivity of TC was studied after 0.5, 2.5, 2.8, and 3.0 years of cultivation and at 28 days of cultivation.

### 2.7. Elicitor Treatments of Target Metabolites

The elicitor effect on the biosynthesis of catechins and alkaloids in LSC-5Y CSC was investigated using two biotic elicitors: methyl jasmonate (MeJA) at concentrations of 5 µM and 10 µM, and an elicitor derived from the fungus *Ciborinia camelliae*. Solutions (500 μL) of MeJA in 50% (*v*/*v*) ethanol (EtOH), 50% (*v*/*v*) EtOH (control), and *C. camelliae* extract were added to the culture medium to achieve the desired final concentration in the cell culture (5 and 10 µM). *C. camelliae* extract was obtained by isolating the fungus from *C. japonica* flowers on potato dextrose agar (DifcoTM, Becton Dickinson, Sparks, MD, USA) medium and culturing it for several months at 25 ± 2 °C in the dark. Mycelium from a 46-day-old culture was harvested, dried at 60 °C for 24 h, ground to a fine powder, and dissolved in Milli-Q water to a concentration of 20 mg/mL. This solution was autoclaved at 121 °C for 15 min and filtered through a filter paper (1242, Filter-Lab, Filtros Anoia, S.A., Barcelona, Spain), and 500 µL of the resulting solution was added to the tea CSC.

All elicitation treatments, including the addition of MeJA solutions and *C. camelliae* extract, as well as a control treatment (addition of EtOH solution and no addition), were analyzed in terms of biomass and target metabolite productivity. Cultures were initiated from the 15-day-old mother CSCs in 125 mL Erlenmeyer flasks with 30% of the flask’s volume as the working volume and 25 g/L as the inoculum size in Ep medium containing 2 mg/L BA, 0.1 mg/L IBA, 0.8 g/L Gln, 0.1 g/L Ser, and 3% sac. Culture conditions were the same as previously described, and elicitor and control solutions were added after 20 days of culture, with an elicitation period of 24 h for all treatments.

### 2.8. Microscopic Observation and Cell Staining

Cell viability was assessed at different stages of growth kinetics by staining tea cells with fluorescein diacetate (FDA), according to a modified protocol based on previous work [19]. Briefly, 10 µL of FDA solution (1.2 mM) was mixed with 0.5 mL of tea CSC. The resulting mixture was incubated in the dark for 5 min, after which the samples were visualized using an epifluorescence microscope (Nikon Eclipse 50i, Nikon Inc., Tokyo, Japan) equipped with a white LED, mercury light sources, and a Nikon digital camera (DS-5M). Viable cells appeared green under fluorescence, and the percentage of viability was calculated by enumerating live cells relative to the total number of cells observed. To ensure accuracy, at least 100 cells were counted in the 3 replicates for each of the treatments.

### 2.9. Biomass Measurements

In all experiments, cell growth was assessed using the gravimetric method, which involved measuring both the initial and final fresh cell weights, as well as the final dry cell weight. The results of all experiments were expressed in terms of dry biomass productivity, measured in grams of cellular biomass per liter of culture. However, in the inoculum density assay, the specific growth rate was analyzed. This parameter was calculated on the basis of fresh biomass using the following formula: (Final fresh weight − Initial fresh weight)/Initial fresh weight/Days. The fresh biomass was dried in an oven at 60 °C until the DW was constant. The DW was recorded and the dry material was ground in a mortar to a fine powder. Dry samples were stored at −80 °C until further chromatographic analysis.

### 2.10. Preparation of Extracts for HPLC Analysis

The extraction method was based on ISO 14502-2:2005 [20] with some modifications. Approximately 0.20 g dry weight of the sample was weighed into extraction tubes, and methanol 70% was added in a ratio of 1:10 (*w*/*v*). The sample was incubated for 10 min at 70 °C in a water bath. The samples were then tempered at room temperature and centrifuged at 4500 rpm. The extraction procedure was repeated twice. The samples were filtered using syringe filters with a 0.45 µm pore diameter polyethersulfone membrane and diluted 1:4 with 70% methanol.

### 2.11. HPLC Analysis

Samples were analyzed by a liquid chromatography–photodiode array detector (HPLC-PDA) using a Varian 920-LC system (Agilent Technologies, Santa Clara, CA, USA) and a Waters SunFire C18 stationary-phase 250 mm length × 4.6 mm i.d. and 5 µm particle size (Scharlau, Barcelona, Spain), following the procedure of Wang et al. [18] with some modifications. The column temperature was adjusted to 35 °C. The solvent mixture contained 1% acetic acid in water (A) and acetonitrile (B), with a flow rate of 1.2 mL/min. The separation was performed using a gradient flow as follows: initial 90% A and 10% B; 20 min—87% B and 13% A; 20 min—70% A and 30% B; 10 min—65% A and 35% B; 1 min—90%A and 10% B. The injection volume was 20 μL and detection was performed using a photodiode array at 280 nm. Peak identification in samples was based on comparison of the UV spectrum and retention time with the authentic standard.

All determinations were performed in triplicate, and data were expressed as mean ± standard error, expressed in terms of productivity (mg of target metabolite per liter of cell culture), except for callus target metabolite content, which is expressed in terms of yield (mg of target metabolite per gram of dry callus). TC content is the sum of all identified catechin monomers; GC, EGC, C, EC, CG, and ECG.

### 2.12. Statistical Analysis

Data were presented as the mean ± standard deviation of at least three replicates of each experiment. Data were analyzed by one-way analysis of variance (ANOVA) and Tukey’s HSD test (<0.05). Statistical analyses were performed using the software R, version 4.3.2 (R Project for Statistical Computing, Vienna, Austria). In addition, the interaction between inoculum density and cell line culture age (1st, 2nd, or 3rd generation) was examined using a two-factor ANOVA analysis.

## 3. Results and Discussion

### 3.1. Selection of LSC-5 Callus Lines under Light Treatment

**Figure 2:**
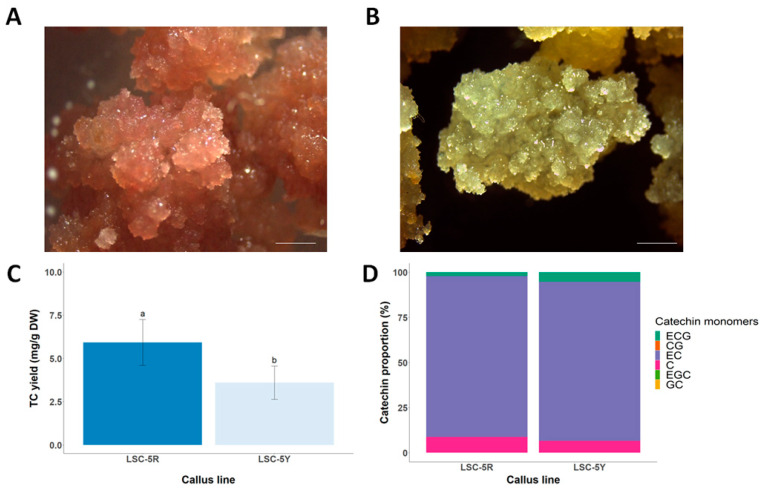
Image of the morphology of LSC-5R (**A**) and LSC-5Y (**B**) callus. Yield of total catechin (**C**), and percentage of each catechin monomer (**D**) in LSC-5R and LSC-5Y callus quantified by HPLC. Scale bar is 5 cm. Different letters (a,b) indicate statistically significant differences between samples (*p* < 0.05).

Selection of callus lines with a higher productivity of secondary metabolites is an important and first step to obtain productive cell lines due to the theory of biochemical heterogeneity in plant callus [21,22]. In this sense, light can act as a selection factor in the tea plant and callus by inducing phenotypic and metabolic changes [9,23]. In in vitro cultures, many studies have observed that light acts as a short-term elicitor (one or more subcultures) of catechins to more than 2 times their content in tea callus by stimulating the synthesis of enzymes involved in the flavonoid biosynthesis pathway [18,23,24]. In tea CSCs, Shibasaki’s study [14] also shows that light increases catechin productivity in four consecutive subcultures. However, light as a long-term selection factor for dark-grown calli has not been investigated. In our work, it is observed that light stimulates a phenotypic change in LSC-5 callus through the formation of reddish (LSC-5R) and yellow areas (LSC-5Y) (Figure 2A,B). This phenotypic change is stable over time when the LSC-5R callus line is cultured in the dark for one year. Interestingly, the LSC-5R callus line has a greater capacity for catechin production of 1.65 times more than the LSC-5Y callus line (Figure 2C,D). Furthermore, an important characteristic of the LSC-5R callus line is its flavanol production capacity, whereas this type of flavonoid is not detected in the LSC-5Y line.

**Figure 3:**
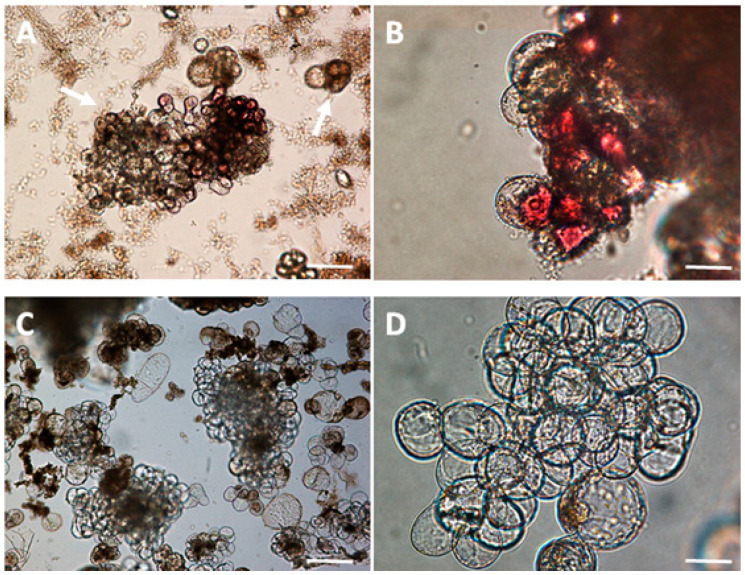
Cell morphology of LSC-5R CSC callus (scale bar at 1 mm, (**A**), scale bar at 50 µm, (**B**)). Cell morphology of LSC-5Y CSC (scale bar at 1 mm, (**C**), scale bar at 50 µm, (**D**). The arrows point to necrotic cells.

In field-grown *C. sinensis* plants, red-purple cultivars were also found to have higher catechin and anthocyanidin content compared to cultivars with green leaves [25]. This could be in line with our results. Thus, the selection of red-purple tea cells under light conditions could be an interesting characteristic trait to induce flavonoid metabolism in a continuous manner over time. However, the disadvantage of this LSC-5R cell line is its inability to establish itself in liquid medium due to the high degree of necrosis after several subcultures (Figure 3). This establishment problem should be studied in future studies since the joint synthesis of flavonols and the flavan-3-ols (catechins type) by the LSC-5R cell line is interesting because both types of compounds may have synergistic effects, for example in antihyperglycemic activity, which may lead to the development of new drug formulations [26]. The necrosis in the red line LSC-5R could be due to the presence of more phenolic compounds, including catechins, compared to the yellow line LSC-5Y [27]. This phenomenon has been observed during cultivation and especially in the establishment of CSCs in different genera of economic importance, such as the genus *Taxus* and *Glycyrrhiza*, and is mainly related to the abundance of phenolic compounds [28]. Oxidation of phenolic compounds by the enzyme polyphenol oxidase (PPO) produces *o*-quinones, which can spontaneously condense and form polymers that cause browning of cultures and cell death.

### 3.2. Effect of PGR Concentration on the Biomass and Target Metabolite Productivity

**Figure 4:**
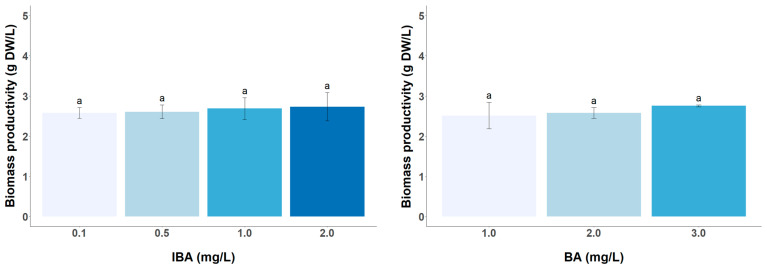
Effect of different concentrations of IBA on biomass productivity of LSC-5Y CSC, supplemented with 2 mg/L of BA (**left**) and different concentrations of BA, supplemented with 0.1 mg/L IBA (**right**). In each graph bars followed by different letters (a) are significantly different (*p* < 0.05).

The type and level of PGRs added to the culture medium play a key role in the balance between cell dedifferentiation, differentiation, and cell growth, but they have also been shown to directly influence the synthesis of secondary metabolites [29,30,31,32]. In *C. sinensis* CSCs, many studies have observed different requirements for the type and concentration of PGRs in the MS medium to achieve optimal growth [12,13]. In all these previous studies, 2,4-D auxin was chosen in combination with a cytokinin (BA or KIN) or with another auxin (IAA), and different concentrations of these phytohormones were used. Only the study by Groover et al. [13] examined the effect of the concentration of BA and 2,4-D on biomass productivity and showed that there was an effect of both hormones on the growth of tea cells. On the contrary, the tea cell line developed in this study (LSC-5Y CSC), grown in Ep medium, does not suffer any type of change in terms of cell growth when varying the concentration of BA and IBA, which is interesting because it allows the selection of the optimal concentration of PGRs to improve the productivity of catechins without sacrificing the biomass productivity (Figure 4). The biomass productivity obtained is not comparable with the results obtained by Grover et al. [13], because the cell growth is expressed as a growth ratio on a dry weight basis. The productivity of dry biomass is lower than in the tea CSC developed by Shibasaki-Kitakawa et al. [14] of around 10 g DW/L, when the culture was started with the same inoculum density of 25 g/L as in the present work.

**Figure 5:**
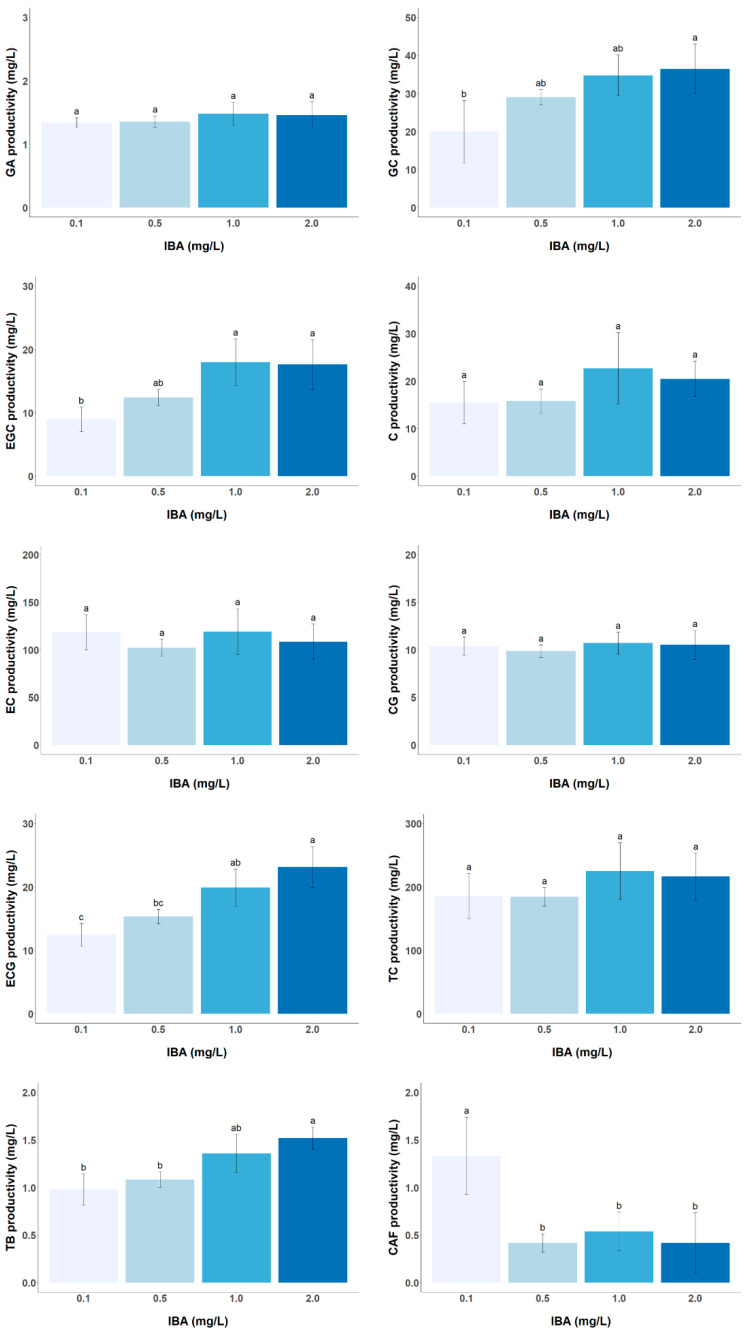
Effect of different IBA concentrations (0.1, 0.5, 1.0, and 2.0 mg/L) in Ep medium supplemented with 2 mg/L BA on the productivity of target metabolites (GA, GC, EGC, C, EC, CG, ECG, TC, TB, and CAF) in LSC-5Y CSC. In each graph bars followed by different letters (a–c) are significantly different (*p* < 0.05).

**Figure 6:**
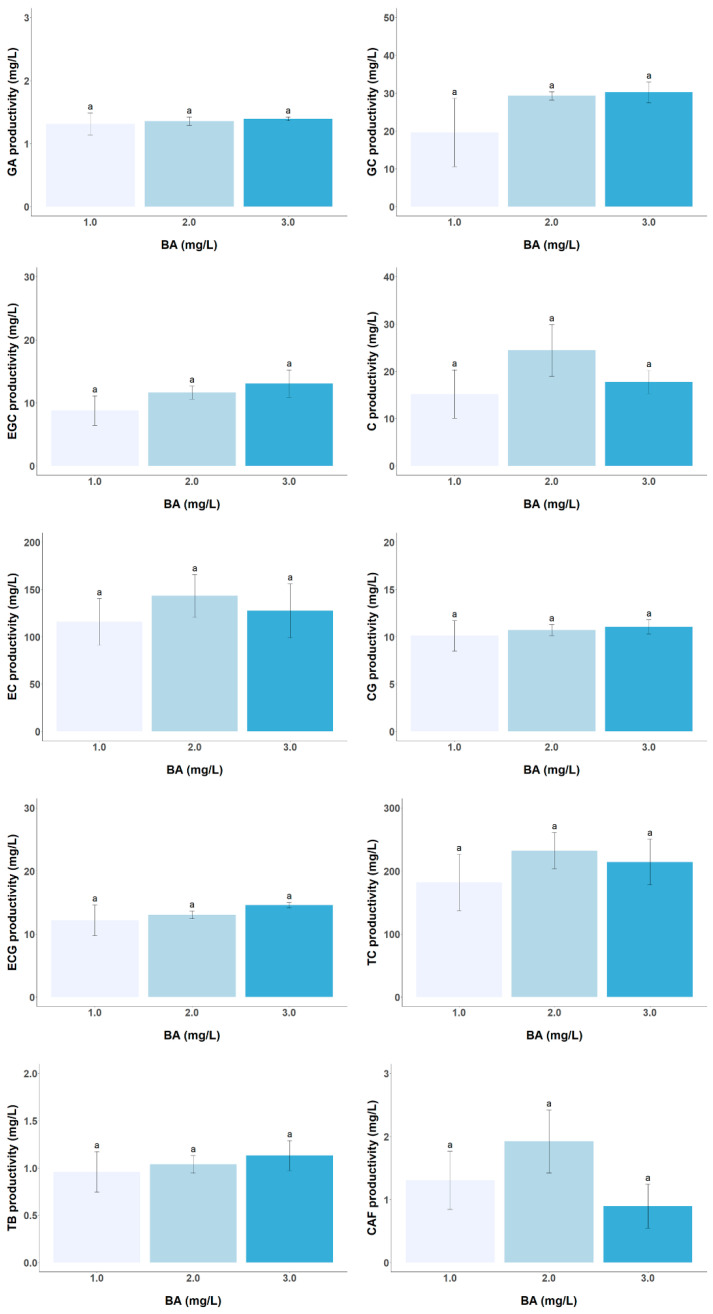
Effect of different BA concentrations (1.0, 2.0, and 3.0 mg/L) in Ep medium supplemented with 0.1 mg/L IBA on the productivity of target metabolites (GA, GC, EGC, C, EC, CG, ECG, TC, TB, and CAF) in LSC-5Y CSC. In each graph bars followed by different letters (a) are significantly different (*p* < 0.05).

The effect of auxin and cytokinin concentrations on catechin productivity in the tea CSC was investigated. The results show a significant influence of the auxin IBA on both catechins (except in EC and CG contents and TC productivity) and alkaloids analyzed, while the cytokinin BA shows no noticeable effects within the concentration range studied (Figure 5 and Figure 6). Increasing the IBA concentration up to 20-fold, from 0.1 to 2.0 mg/L, leads to a gradual increase in the productivity of the two trihydroxycatechins (GC and EGC) as well as ECG by approximately 1.82, 1.96, and 1.86-fold, respectively. Previous research has shown that low auxin concentrations of the order of 0.5 mg/L inhibit the formation of phenolic compounds that share biosynthetic pathways with catechins (shikimate and flavonoid pathways), such as isoflavones in *Genista tinctoria* callus or anthraquinones in *Polygonum multiflorum* CSC [31,33]. It appears that in this tea CSC, IBA concentrations between 0.1 and 0.5 mg/L may inhibit flavan-3-ols formation. Thus, higher auxin concentrations (>0.1 mg/L) promote TB accumulation but inhibit CAF formation, suggesting a possible inhibition of TB methylation to form CAF by the enzyme caffeine synthase [34]. Based on these results, a medium consisting of Ep supplemented with 2 mg/L of BA and 1 mg/L of IBA could be used for catechin production. However, if this medium is to be used for both cell growth and catechin production in a single-phase culture system, the long-term stability of cell growth and productivity of target metabolites would need to be further investigated.

### 3.3. Effect of Inoculum Size on Biomass and Target Metabolite Productivity

**Figure 7:**
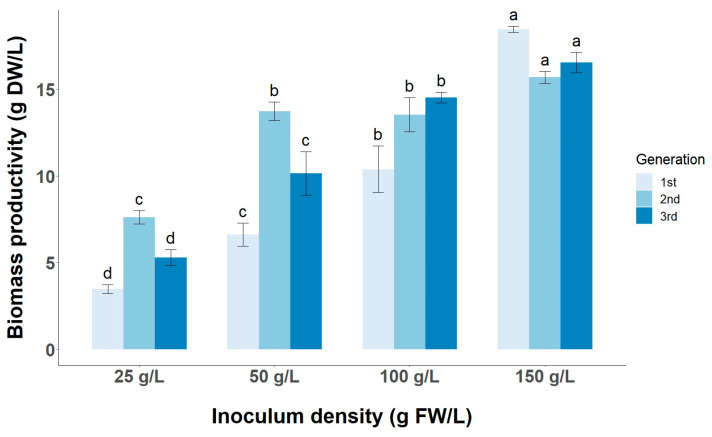
Effect of inoculum size (25, 50, 100, and 150 g/L) on biomass productivity during three consecutive CSC subcultures of tea (generations). Different letters (a–d) in the same inoculum density and generation indicate statistically significant differences (*p* < 0.05).

**Figure 8:**
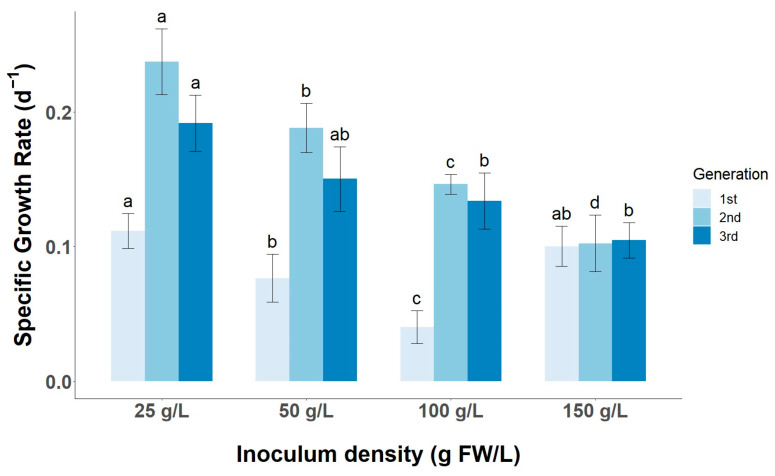
Effect of inoculum size (25, 50, 100, and 150 g/L) on the specific growth rate during three consecutive CSC subcultures of tea (generations). Different letters (a–d) in the same inoculum density and generation indicate statistically significant differences (*p* < 0.05).

Understanding how the inoculum size affects the cell growth and the production of target metabolites is essential for scaling up cultures in pilot- and industrial-scale bioreactors. Furthermore, it is important to select not only the inoculum density at which the faster growth occurs (specific growth rate) but also at which optimal or competitive biomass productivity is achieved at an industrial scale. A gradual increase in biomass productivity is observed with increasing inoculum size, reaching a maximum of 18.47 ± 0.17 g DW/L after 21 days of culture in the CSC started with 150 g/L in the first generation (Figure 7). This productivity is higher than that found in the studies by Shibasaki and Grover [13,14,15,16], with productivities of around 10 g DW/L, although the cultures were started at 25 g FW/L and 1 g DW/L of inoculum density. On the other hand, the cell line shows an optimal specific growth rate at low inoculum densities of 25 g/L and 50 g/L of the order of 0.20 d^−1^ (Figure 8). In contrast, Ying Qin et al. [16] showed that higher inoculum densities of the order of 80 g/L must be used to achieve a specific growth rate of about 0.25 d^−1^ in tea CSC in MS medium when cells are harvested after 16 days of culture. The differences in biomass productivity and specific growth rate may be due to the use of MS medium supplemented with different PGRs, pH of the culture medium, working volume and Erlenmeyer flask volume, inoculum density, light regime, or degree of agitation [12,13,35].

**Figure 9:**
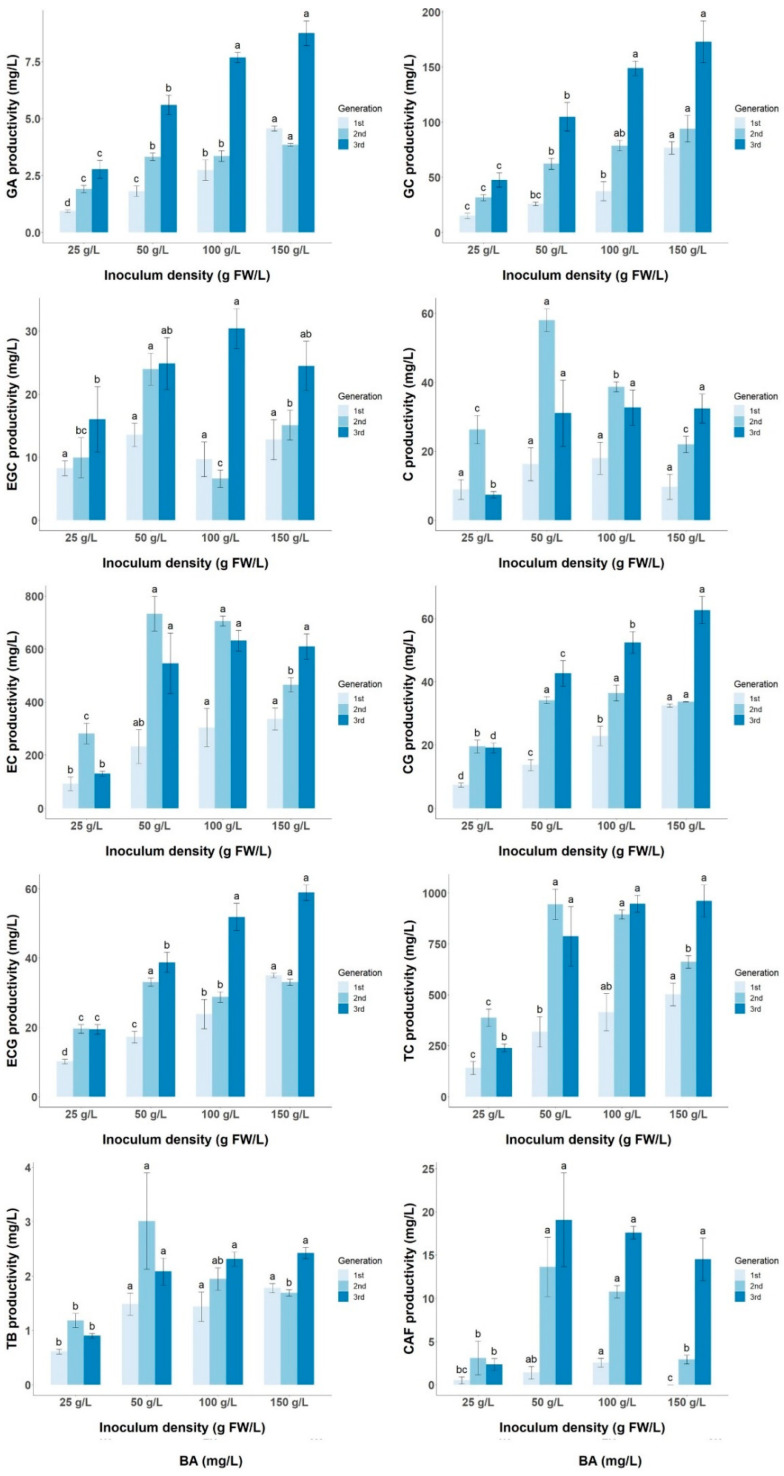
Effect of inoculum size (25, 50, 100, and 150 g/L) on the productivity of target metabolites (GA, GC, EGC, C, EC, CG, ECG, TC, TB, and CAF). In each graph bars followed by different letters (a–d) in the same inoculum density and generation indicate statistically significant differences (*p* < 0.05).

In addition to its effect on cell growth, inoculum density plays a crucial role in modulating the accumulation of secondary metabolites [32,36]. Several studies have demonstrated the stimulatory effect of high inoculum size on enzymes within the phenylpropanoid pathway, thereby increasing the yield and productivity of various phenolic compounds such as anthocyanins in *Perilla frutescens* CSC and flavones in *Cajanus cajan* CSC, as well as in the accumulation of other secondary metabolites such as ginseng saponins in *Panax ginseng* CSC and taxol in *Taxus yunnanensis* CSC [21,36,37,38]. However, the optimal inoculum density to optimize metabolite productivity varies between species; for example, in *Perilla frutescens,* the optimal inoculum density is 50 g FW/L, in *Cajanus cajan* CSC, it is 3% *w*/*v*, in *Taxus yunnanensis,* it is 200 g FW/L, and in *Panax ginseng,* it is 6 g DW/L. In this study, the inoculum density had a negligible effect on the yield of GA, GC, ECG, and CG, while it had a significant effect on the yield of C, EGC, EC, CAF, and TB, so the inoculum density dropped below 25 g/L and 150 g/L reduced the yield of these metabolites. Analyzing the metabolite yield and the cell biomass productivity together, it can be observed that intermediate inoculum densities of 50 and 100 g/L increased the productivity of C, EGC, EC, and CAF, whereas for GA, GC, CG, and ECG, the productivity increased with increasing inoculum density (Figure 9). In the case of TC productivity, there was a significant difference between the 25 g/L inoculum density and the other treatments, with a productivity increase ranging from 2.44 to 4.01 times over three generations. The maximum TC productivity reached 960.61 ± 79.03 mg/L at an inoculum density of 150 g/L, which is slightly lower than the productivity of 1500 mg/L obtained by Shibasaki-Kitakawa et al. [14], although in this work, the total flavonoids are quantified and not the sum of monomeric catechins.

**Figure 10:**
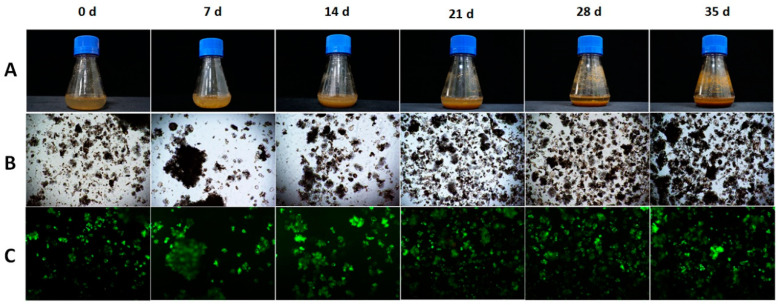
Images of an Erlenmeyer flask of LSC-5Y CSC culture at different days (**A**), cells observed under the microscope (**B**), and viable cells stained with FDA under the microscope (**C**).

**Figure 11:**
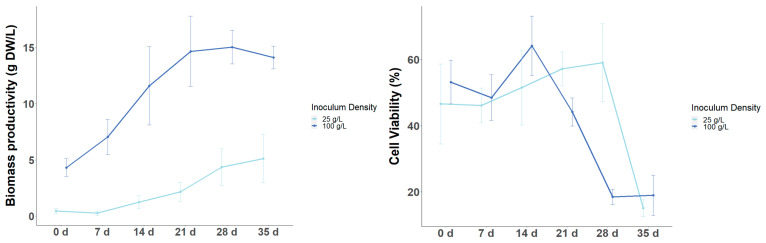
Biomass productivity (**left**) and cell viability (**right**) at different days of LSC-5Y CSC culture (0, 7, 14, 21, 28, and 35 days of culture).

The variation in the productivity of target metabolites as a function of inoculum size may be due to the fact that the initial cell biomass affects the growth kinetics, as it influences the duration of the growth phases (lag, log, stationary, and death phases), and it is known that these growth phases are associated with the biosynthesis of secondary metabolites [12]. In the case of tea CSCs, Shibasaki-Kitakawa et al. [14] and Muthaiya et al. [12] showed that catechins are biosynthesized and accumulate more at the end of the exponential phase or in the stationary phase compared to the lag phase and the early logarithmic phase. Furthermore, Shibasaki-Kitakawa’s [14] work showed a decrease in flavonoid content in the cell death phase. Therefore, when the growth kinetics of LSC-5Y CSC are initiated at two different inoculum densities (25 and 100 g FW/L), no dormancy is observed in the cell culture initiated at the high inoculum density (100 g/L), and a dormancy of 7 days is observed in the culture initiated at the low inoculum density (25 g/L) (Figure 10 and Figure 11). The stationary phase is reached after 21 days in the culture started at 100 g/L, whereas the culture started at 25 g/L is reached after 28 days, and in both treatments, there is a reduction in cell viability from around 50% of live cells to around 20% (Figure 10B). These differences may contribute to the different yield and productivity of target metabolites from the cell culture started with different inoculum sizes.

### 3.4. Short-Term and Long-Term Stability of TC Productivity in LSC-5Y Cell Line

**Figure 12:**
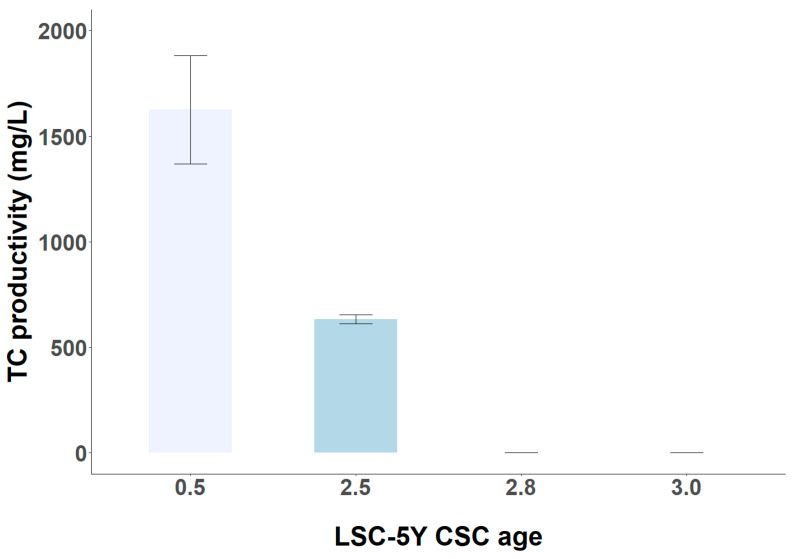
Long-term stability of TC productivity in LSC-5Y CSC with increasing cell line age (0.5, 2.5, 2.8, and 3.0 years).

The stability of target metabolite productivity by plant CSCs is the main bottleneck in the commercialization of valuable bioactive products for the cosmetic, pharmaceutical, or food industries. The variation in the yield and target metabolite productivity has been observed in different plant species and classes of secondary metabolites such as the production of anthocyanins in *Vitis vinifera* CSC, of taxol in Taxus CSCs, and of alkaloids in transgenic *Cantharanthus roseus* CSCs [39,40,41]. In the case of *C. sinensis* CSCs, the LSC-5Y cell line has the capacity to produce catechins for 2 and a half years, after which it no longer has the capacity to produce catechins (Figure 12). During this time, the productivity of catechins by LSC-5Y CSC decreases with the age of the cell line, from a productivity of 1625.48 ± 102.91 mg/L at 0.5 years after its establishment in liquid medium to a productivity of 633.50 ± 8.53 mg/L of TC after 2.5 years of culture. The productivity of the LSC-5Y cell line after 0.5 years (low light intensity and no elicitation treatment) was higher than the flavonoid productivity obtained by Shibasaki-Kitakawa et al. [14] after using light as an abiotic elicitor during several subcultures, in the order of 1500 mg/L. In addition, the variability of TC productivity was also studied over 3 months of culture with an age of the cell line of 2.08 years, observing high coefficients of variation in TC productivity in this period, of the order of 45.75, 47.67, 39.27, and 32.80%, when the culture was started with an inoculum amount of 25, 50, 100, and 150 g/L, respectively. This instability in the target metabolite productivity may be due to the heterogeneity of the source of explant material, resulting in a heterogeneity of dedifferentiated cells with different catechin synthesis capacities, genetic and epigenetic instabilities, changes in the pattern of cellular aggregates, and inefficient cell selection during subculturing [22,39,40,41]. Furthermore, the interaction of factors such as inoculum density and cell line age on biomass productivity, specific growth rate, and TC productivity (all *p*-values < 0.05) shows the complexity of controlling cultivation factors to optimize TC production, which requires constant monitoring of physical, chemical, and biological parameters, and continuous implementation of improvements in this bioprocess system is necessary [42].

### 3.5. Effect of Different Elicitors on Catechin Productivity by LSC-5Y CSC

**Figure 13:**
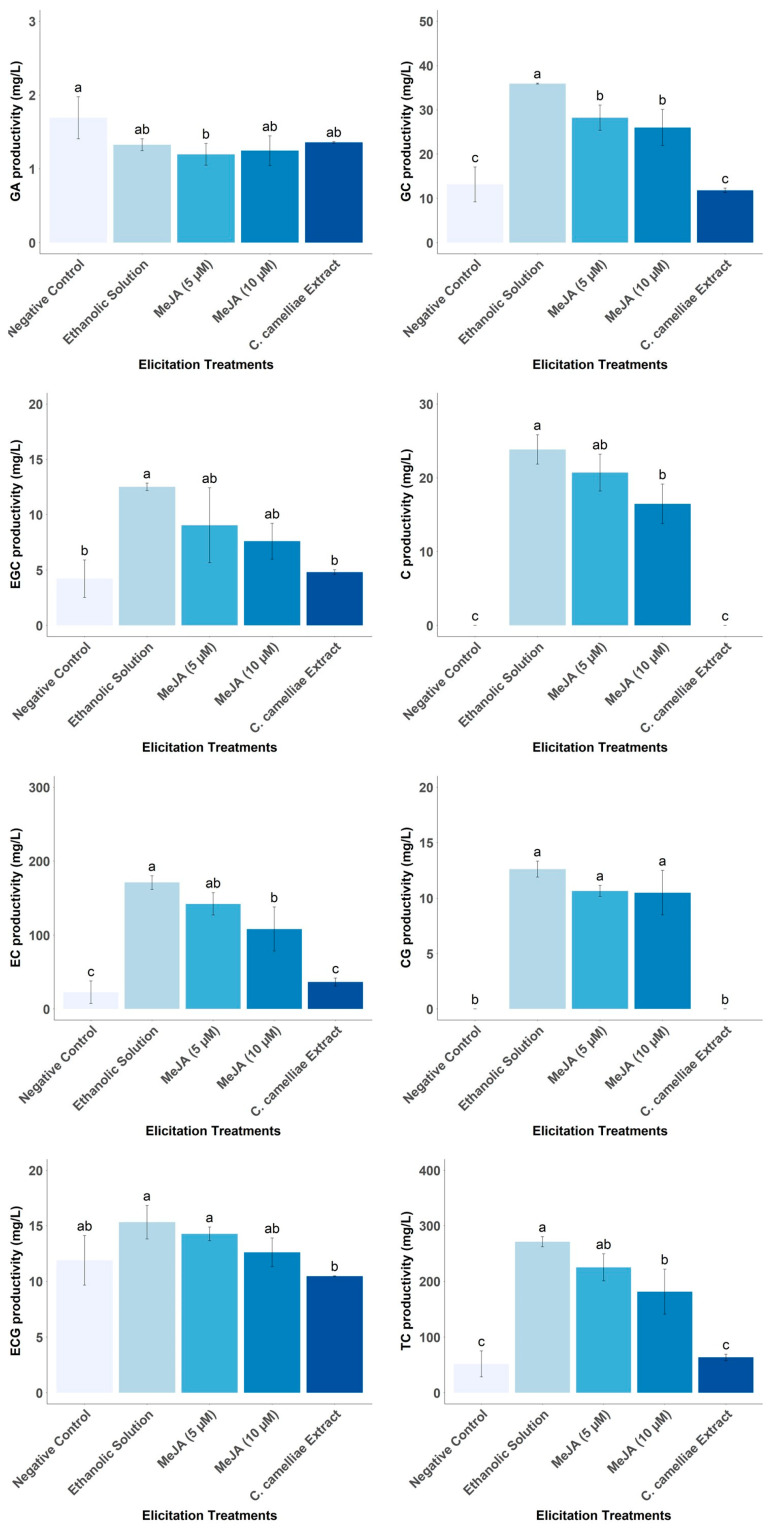
Effect of different elicitor treatments (MeJA and *C. camelliae* extracts) on target metabolite productivity of LSC-5Y CSC, compared to control treatments. In each graph bars followed by different letters (a–c) indicate statistically significant differences (*p* < 0.05).

The main limitation of plant CSCs is the low yield and productivity of the bioactive compounds of interest. In the case of *C. sinensis* CSC, the yield of catechins is high and can exceed the yield of field leaves of about 150 mg/g DW [12,13,14]. Since the beginning of the 21st century, different studies focused on tea CSCs have investigated the elicitation techniques to increase the yield and productivity of catechins, which consist of the addition of precursors of the biosynthesis pathway [12], the application of light as an abiotic elicitor [14], and the addition of plant hormones (MeJA, salicylic acid, gibberellin 3, ethylene, and abscisic acid) as biotic elicitors [16]. Using these tests, it was possible to increase the yield or productivity of total phenolic compounds or total catechins between 1.5 and 10 times. The effect of an exogenous biotic elicitor (derived from the fungus *C. camelliae*) on CSCs in tea was investigated for the first time. However, it was observed how the ethanolic solution used as a control and the treatments with MeJA (5 and 10 µM) exerted an elicitor effect on the production of catechins between 3.50 and 5.24 times compared to the control (Figure 13). The increase in catechin levels exceeded the 2.2-fold increase in total polyphenols reported by Qin et al. [16] on elicitation of tea CSCs with the addition of the endogenous biotic elicitor abscisic acid (200 µmol/L) for two days. The total polyphenol productivity reported by Qin et al. [16] was 397.05 mg/L, slightly higher than the results of elicitation treatments with EtOH and MeJA (181.51 and 228.67 mg/L, respectively). However, our results quantify total catechins as the sum of catechin monomers, excluding other phenolic compounds such as gallic acid or flavonols, whereas these results were reported as total phenolics. On the contrary, although it has been shown that the fungus *C. camelliae* is a specialist fungus of the genus *Camellia* and that it forms compatible interactions with the flower of *C. sinensis* [43], no catechin-eliciting effect was observed from the aqueous solution derived from the mycelium of this fungus grown in vitro.

**Figure 14:**
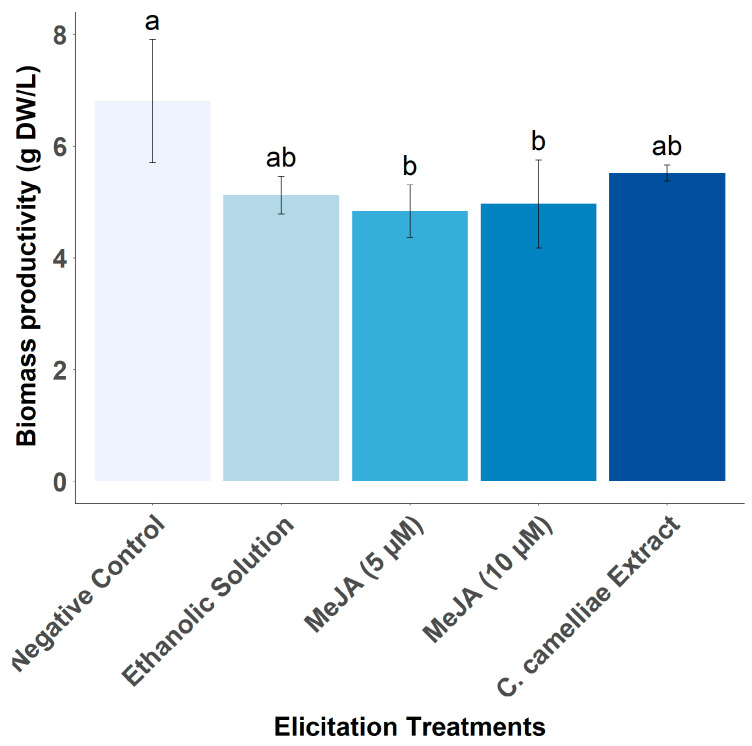
Effect of different elicitor treatments (MeJA and *C. camelliae* extracts) on biomass productivity of LSC-5Y CSC, compared to (negative and positive, ethanol) control treatments. Different letters (a,b) indicate statistically significant differences between samples (*p* < 0.05).

The addition of MeJA has a negative effect on the growth of tea CSC (Figure 14), in agreement with previous studies in other cell lines [44,45]. Interestingly, the ethanolic solution favors the increase in the productivity of GC, EGC, C, EC, CG, and TC, as do the MeJA solutions (5 and 10 μM), except for EGC. For these catechins, the elicitor effect is higher with ethanol treatment than with MeJA. As the MeJA solutions are prepared with 50% ethanol, it is possible that the real elicitor is not the MeJA molecule, but the ethanol, and it is possible that MeJA has a negative effect on the synthesis and/or accumulation of these molecules of interest. Ethanol is an alcohol that can be produced endogenously in plants by fermentation because of anaerobic conditions or can be applied exogenously to plants [46,47]. Previous studies have shown that exogenous application of ethanol induces salt stress tolerance in *A. thaliana*, rice, and soybean and chilling stress tolerance in rice [47,48,49]. The mechanisms associated with ethanol-induced stress tolerance are the prevention and/or delay of the destruction of photosynthetic pigments such as chlorophyll and carotenoids, the up-regulation of genes involved in ROS-scavenging activity, mainly transcription factors and genes encoding antioxidant enzymes. These mechanisms may explain why ethanol acts as an elicitor of catechins in tea CSCs since it has been shown that chlorophyll can be involved in the regulation of individual catechins, increasing their concentrations in leaves [50]. Furthermore, it has been extensively demonstrated that the biosynthesis of different catechin monomers is induced during different types of abiotic stress and has a protective activity through its ability to scavenge reactive oxygen species (ROS) generated during stress situations [6].

In plant CSCs, ethanol has often been used as a solvent for MeJA in elicitation assays, and several studies have investigated its eliciting effect as a negative control [44,45,51], although other studies do not specify whether its effect was tested [16]. The effect of this molecule has been shown to have a negative effect on the anthocyanin yield in *Vitis vinifera* [51] and does not cause any kind of variation in the content of different phenolic compounds in different cell lines [44,45]. In this case, EtOH may be an easily available, cheap, and non-toxic catechin elicitor and should be considered for future studies.

## 4. Conclusions

The effect of different cultivation factors (concentration of PGRs, inoculum density, short and long-term stability of the culture, light exposure, and effect of addition of elicitors), key points in the implementation of bioprocesses on an industrial scale, on the catechin monomers production from *C. sinensis* cell lines was studied. The tea cell line, in Ep medium containing 2.0 mg/L of BA and 0.1 mg/L of IBA, had the ability to produce the main catechin monomers (except for EGCG and GCG) up to two and a half years of growth, after which a drastic reduction its flavonoid productivity was observed. Different cultivation factors, such as the concentration of PGRs or the different inoculum densities affected the catechin monomers in different ways. It is therefore necessary to select the target catechin monomers of interest before designing the culture conditions to be used. The use of IBA auxin concentrations higher than 0.5 mg/L may favor the accumulation of minor catechins such as trihydroxycatechins (GC and EGC) and ECG, and the use of intermediate inoculum densities (50 and 100 g/L) may favor the improvement of the productivity of simple catechins (C and EC), EGC, and the alkaloid CAF. Furthermore, due to the instability of the LSC-5Y cell line, in the long and short term, it would be interesting to test the effect of elicitors such as ethanol at different moments in the life of the cell line to check if a reduction in coefficient of variation of TC productivity could be achieved, thus making the production process more viable. Based on the results of the productivity of the culture compared to plants, future work related to the selective production of catechins of interest to the cosmetic, pharmaceutical, and food industries is proposed. Further studies are needed to improve the stability of the cell line and to be able to scale up to the industrial level.

## Figures and Tables

**Table 1:** Chemical and reagent information.

Chemical Name	Formula	CAS	Catalog Number	Company
Acetic acid	C_2_H_4_O_2_	[64-19-7]	A465250	Fisher-Scientific (Geel, Belgium)
Acetonitrile	C_2_H_3_N	[75-05-8]	A998SK	Fisher-Scientific (Geel, Belgium)
6-Benzylaminopurine	C_12_H_11_N_5_	[1214-39-7]	B0904	Duchefa Biochemie (Haarlem, The Netherlands)
Caffeine	C_8_H_10_N_4_O_2_	[58-08-2]	10263800	Fisher-Scientific (Geel, Belgium)
(+)-Catechin	C_₁₅_H₁₄O₆	[154-23-4]	0976 S	Extrasynthese (Genay, France)
(−)-Catechin gallate	C₂₂H₁₈O₁₀	[130405-40-2]	0972 S	Extrasynthese (Genay, France)
2,4-Dichlorophenoxyacetic acid	C_8_H_6_Cl_2_O_3_	[94-75-7]	D7299	Sigma-Aldrich (Merck, Darmstadt, Germany)
6-γ,γ-Dimethylallylamino purine	C_10_H_13_N_5_	[2365-40-4]	D0906	Duchefa Biochemie (Haarlem, The Netherlands)
(−)-Epicatechin	C₁₅H₁₄O₆	[490-46-0]	0977 S	Extrasynthese (Genay, France)
(−)-Epigallocatechin	C₁₅H₁₄O₇	[970-74-1]	0979 S	Extrasynthese (Genay, France)
(−)-Epigallocatechin gallate	C₂₂H₁₈O₁₁	[989-51-5]	0981 S	Extrasynthese (Genay, France)
(−)-Epicatechin gallate	C₂₂H₁₈O₁₀	[1257-08-5]	0978 S	Extrasynthese (Genay, France)
Gallic acid	C_7_H_6_O_5_	[149-91-7]	398225	Sigma-Aldrich (Merck, Darmstadt, Germany)
(+)-Gallocatechin	C₁₅H₁₄O₇	[970-73-0]	0990 S	Extrasynthese (Genay, France)
(−)-Gallocatechin gallate	C₂₂H₁₈O₁₁	[4233-96-9]	0974 S	Extrasynthese (Genay, France)
Indole-3-butyric acid	C_12_H_13_NO_2_	[133-32-4]	I0902	Duchefa Biochemie (Haarlem, The Netherlands)
Kinetin	C_10_H_9_N_5_O	[525-79-1]	K0905	Duchefa Biochemie (Haarlem, The Netherlands)
Methanol	CH_4_O	[67-56-1]	10675112	Fisher-Scientific (Geel, Belgium)
Methyl jasmonate	C_13_H_20_O_3_	[39924-52-2]	392707	Sigma-Aldrich (Merck, Darmstadt, Germany)
Theobromine	C_7_H_8_N_4_O_2_	[83-67-0]	11493930	Fisher-Scientific (Geel, Belgium)
Theophylline	C_7_H_8_N_4_O_2_	[58-55-9]	10712481	Acros Organics (Geel, Belgium)
Trans-zeatin	C_10_H_13_N_5_O	[1637-39-4]	Z0917	Duchefa Biochemie (Haarlem, The Netherlands)

## Data Availability

The original contributions presented in the study are included in the article, further inquiries can be directed to the corresponding author.
